# Implications of the Westernized Diet in the Onset and Progression of IBD

**DOI:** 10.3390/nu11051033

**Published:** 2019-05-08

**Authors:** Fernando Rizzello, Enzo Spisni, Elisabetta Giovanardi, Veronica Imbesi, Marco Salice, Patrizia Alvisi, Maria Chiara Valerii, Paolo Gionchetti

**Affiliations:** 1 Department of Medical and Surgical Sciences, University of Bologna, Via Massarenti 9, 40138 Bologna, Italy; fernando.rizzello@unibo.it (F.R.); elisabett.giovanardi@studio.unibo.it (E.G.); veronica.imbesi@studio.unibo.it (V.I.); marco.salice@hotmail.com (M.S.); chiaravalerii@hotmail.it (M.C.V.); paolo.gionchetti@unibo.it (P.G.); 2 Department of Biological, Geological and Environmental Sciences, University of Bologna, Via Selmi 3, 40126 Bologna, Italy; 3 Pediatric Unit, Maggiore Hospital, Largo Bartolo Nigrisoli, 2, 40133 Bologna, Italy; Patrizia.alvisi@ausl.bologna.it

**Keywords:** Inflammatory Bowel Disease (IBD), Mediterranean Diet, Western-style Diet (WSD), Nutritional Approach

## Abstract

Inflammatory bowel diseases (IBD) are currently considered multifactorial pathologies in which various combined environmental factors act on a genetic background, giving rise to a chronic inflammation of the gastrointestinal tract. Among the various environmental factors, it now seems clear that the diet plays the major role in IBD onset and progression. Several clinical studies have attempted to understand the impact of diet in the development and progression of these diseases in order to establish useful guidelines for their management. However, the modest and sometimes contradictory results did not lead to the definition of shared dietary suggestions. On the other hand, food fads and recommendations based on anecdotal episodes are often followed by IBD patients to improve their diet. This review provides a critical overview of existing data on the role of diet as a risk factor for IBD. The methodology used was that of analyzing the results of clinical studies conducted on diet and IBD over the last 12 years through PubMed, as well as analyzing the most relevant studies on nutrients and their possible roles in IBD through the knowledge of the mechanisms by which they can modulate the microbiota or the intestinal physiology.

## 1. IBD and Environmental Risk Factors

The etiology of inflammatory bowel disease (IBD) remains unclear. The most accredited theory is the interplay between genetic susceptibility and exposure to environmental risk factors, such as pollution, stress, diet, pathogen infections, antibiotic treatment and cigarette smoking, also known as the exposome paradigm [1]. Many studies evidence a rise in both the incidence and prevalence of IBD in traditionally low-incidence areas, such as southern Europe, Asia and most developing countries where Western lifestyle and nutritional habits are progressively being adopted [2]. The prevalence of IBD has also increased significantly in the pediatric population [3].

Many studies have concluded that people emigrating from low-prevalence regions, such as Asia, to high-prevalence countries like northern Europe are at greater risk of developing IBD. This is especially true for first-generation immigrant children who show similar IBD incidence and prevalence patterns to the indigenous population [4].

These findings would seem to reduce the importance of a genetic predisposition to IBD and incriminate major lifestyle changes for the rising IBD rates observed in developing countries, in immigrant populations moving to high-prevalence countries, and children in developed countries. These considerations suggest that environmental factors significantly modify the expression of Crohn’s Disease (CD) and Ulcerative Colitis (UC). IBD would therefore appear to be a worldwide disease linked to environmental factors such as the diet, lifestyle and pollutants prevalent in industrialized urban societies [4,5].

Diet is a key environmental factor. It plays a major role in the homeostasis of the gut microenvironment, influencing the gut’s microbial composition and functioning, the gut barrier, host immunity and the gut physiology by regulating gut hormone release [6,7]. The diet in the developed “Western” world is very different from the traditional diet of previous generations when the prevalence of IBD was considerably lower. The most radical change has been the switch from a plant-based to an animal-sourced diet. This radically modified the gut microbiota and microbiome decreasing the amount of bacteria involved in fiber degradation, such as *Prevotella and Treponema*. [8,9]. Another important change is the increased presence of xenobiotics in food and especially in meat. Xenobiotics are chemical substance that are not naturally produced or expected to be present within the organism and that mainly derive from environmental pollution or from pesticide, widely used in conventional agriculture. Xenobiotics can undergo the bio-magnification process in intensively farmed animal meat. In fact, changes in the gut microbiome and xenobiotic metabolism have been found in children with Crohn’s disease [10].

Recent data suggest that another important change brought about by the Western diet is an overall higher calorie intake, especially from sugar, refined carbohydrates, animal proteins and ultra-processed foods. The role of these latter in gut homeostasis is still under investigation [11], and there are not in-depth studies linking ultra-processed foods and IBD, however, it is becoming increasingly evident that some food additives have negative effects on the gut microbiota.

In conclusion, diet may well play a key role in both the pathogenesis and clinical course of IBD. As a result, diet should become an effective tool to optimize the efficacy of the conventional treatment of these diseases. 

## 2. The Microbiota and IBD

The human gut is colonized by a specific and dynamic subset of trillions of microorganisms. Fundamental for our body functions, these microorganisms weigh about 1 kg—significantly more than all the cells of the human body—and contain more than 100 times as many genes as the human genome. They constitute the so-called ‘gut microbiota’ of the host’s gastrointestinal tract [12].

The human microbiota comprises different types of microorganisms, including bacteria (prokaryotes), archaea, fungi, protists, and viruses. Its composition differs significantly in the different regions of the gut. Indeed, the displacement of microbial communities within the intestinal tract can sometimes give rise to pathologies such as the small intestinal bacterial overgrowth (SIBO) syndrome [13].

The gut microbiota and its host exist in a symbiotic relationship. Together they form a sort of “super-organism” with a wide range of functions. These include digesting substrates, producing nutrients, developing and regulating the immune system, preventing the growth of harmful microorganisms by forming a barrier against colonization or invasion, and detoxifying certain xenobiotics [14]. An adequate biodiversity of the microbiota plays an important role not only for the correct functions of the intestine but also in other organs and systems, such as the enteric and central nervous system [15]. Brain and microbiota interactions have led to the recognition of a new term called "microbiota-gut-brain axis". This term refers to a two-way communication system that allows intestinal microbes to change the physiology of the brain and the brain to modulate the intestinal functions. The evidence of this two-way communication comes from multiple studies that have shown that stressful events in an individual’s life can alter the composition of the intestinal microbiota, as well as reciprocally, the infection of intestinal pathogenic bacteria can induce anxiogenic behavior in mice [16,17]. Moreover, impaired responses of the central nervous system to stressors have also been clearly demonstrated in patients with microbiota alterations due to active Crohn’s disease [18].

**Figure 1:**
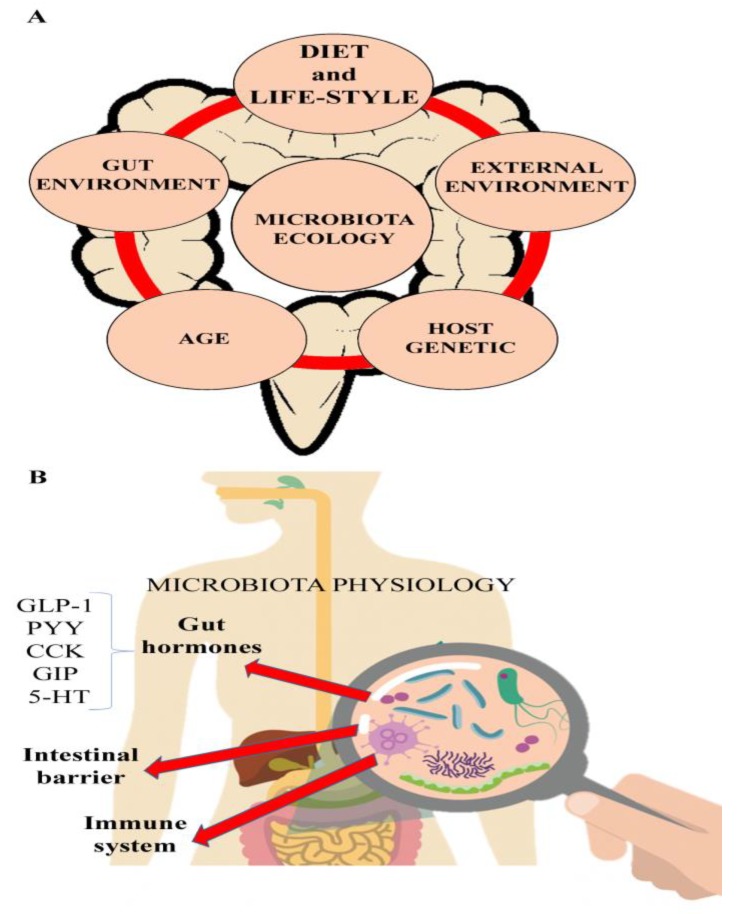
Factors implicated in the modulation of the microbiota ecology (**A**) and complex microbiota interactions with the gastrointestinal physiology (**B**). PYY, Peptide YY; GLP-1, Glucagon-like peptide-1; CCK, Cholecystokinin; GIP, Gastric inhibitory polypeptide; 5-HT, 5-hydroxytryptamine or Serotonin.

Another characteristic of the gut microbiota is its inter-individual variability due to genetic predisposition and factors such as environmental microbes, geography, climate, sanitation, pollution, diet and lifestyle (Figure 1). It follows that the composition of an individual’s microbiota changes continuously in a mutualistic response to extrinsic and intrinsic variables. Excessive alterations of the gut microbiota lead to an intestinal imbalance between protective and harmful bacteria, called dysbiosis. This condition seems to be responsible for the inadequate response of the immune system to both external and intestinal epitopes, and may contribute to the loss of oral tolerance. This imbalance can have serious effects during infancy and early childhood when the microbiota is still maturing [19]. Dysbiosis may also be a leading contributory cause of several gut microbiota-related diseases, including IBD [20,21].

While many studies have clearly demonstrated the presence of severe intestinal permeability alterations during the active phases of IBD, permeability seems to recover during the remission phase. It should be noted, however, that modest alterations in intestinal permeability are difficult to be measured *in vivo* [22].

IBD are associated with alterations in the composition of the intestinal microbiota, namely a significant reduction of bacterial diversity, an increase in several pro-inflammatory components called “pathobionts”, such as Proteobacteria and Actinobacteria, and the depletion of “health-promoting” anti-inflammatory bacterial genus, such as *Faecalibacterium* and *Roseburia* [23].

During the acute phases of IBD, altered intestinal permeability causes commensal bacteria and microbial antigens to translocate from the lumen to the submucosa, giving rise to local and systemic inflammation. These translocations activate the immune cells, which in turn release numerous pro-inflammatory cytokines, activating an inflammatory loop and inducing chronic intestinal inflammation [24].

The microbiota’s plasticity allows the gut microbiome to respond rapidly to changes in dietary patterns. The fermentable carbohydrates, protein, fat, and sugars ingested modulate the levels of beneficial and harmful microbes, toxic microbial metabolites and protective metabolites, such as short-chain fatty acids (SCFAs) [25].

Dietary fibers, polysaccharides, sugars (i.e. lactose) and peptides that are not digested by host enzymes in the upper gut are partly degraded by the gut microbiota in the cecum and colon through a process called fermentation. The major products of fiber fermentation are SCFAs, in particular, acetate, propionate, and butyrate [26]. SCFAs play important multifactorial roles in energy homeostasis, host immune function [25,27] and general gut physiology [25,28] (Table 1). As a result, the amount of SCFAs synthesized, especially in the colon, has been adopted as an indirect measure of microbiota health [28]. High salt consumption also affects the gut microbiome of both mice and humans by lowering the intestinal survival of Lactobacillaceae [29].

The modulation of intestinal microbiota can be also implemented through probiotics. Probiotics have been defined by the World Health Organization (WHO) as "live microorganisms which, when administered in adequate quantities, confer health benefits to the host". They can play beneficial effects on the digestive system, through multiple mechanisms including interference with potential pathogenic microorganisms, improvement of the barrier function, immunomodulation and the modulation of neurotransmitter synthesis [30,31]. The impact of probiotics on IBD has been the subject of numerous studies. Data derived from studies on animal IBD models showed a high potential of different bacterial strains in modulating the clinical course of experimental IBD. Instead, on humans the experimental results showed little evidence of efficacy on Crohn’s disease (CD), modest data on Ulcerative Colitis (UC) but a good support for patients affected by pouchitis, the inflammation of the ileal pouch which is created in the management of IBD patients [32].

It has recently been demonstrated that several dietary components can alter the intestinal microbiota. These include xenobiotics belonging to the group of environmental contaminants, but also additives normally used for the preparation of ultra-processed foods. The xenobiotics capable to have negative effects on the microbiota include organochlorine pesticides [33], glyphosate [34] and perfluorooctanesulfonic acid [35]. All these components have been found in human breast milk, and may therefore negatively modulate the microbial ecology of babies whose unstable microbiota could rapidly develop into a permanently altered gut environment [35]. The effect of food additives added to ultra-processed foods will be discussed in detail below. 

Studies of IBD-associated microbiome have evidenced the important role played by the gut environment in the manifestation of IBD. It has also been observed that among the environmental factors associated with microbiota development and alterations, diet plays a key role in modulating the composition and functions of the microbiome and epithelial barrier, and may even influence epigenetic changes. It follows that the food may deeply modulate the microbiome-gut axis and therefore it may represent an important therapeutic tool to counteract dysbiosis and modify the natural history of IBD, bearing out the motto “*We are what we eat*” [7].

## 3. Evolution of the Human Diet

The first dramatic change in human nutrition occurred about 10,000 years ago during the Neolithic age with the transition of the hunter/gatherer society to one based on agriculture and livestock farming. This change profoundly modified man’s diet. Instead of a wide range of different foods that changed with the seasons and with the migratory patterns of animals, our diet became restricted to a few domesticated plants and farmed animal species. For the human metabolism this meant a dramatic change in glycemic load, fatty acid composition, macro- and micro-nutrient concentration and total fiber content. Indeed, in evolutionary terms, this dietary change is so recent that the human species has not had enough time to adapt its gastro-intestinal physiology [36]. Many foods common in the new diet were completely absent in the hunter/gatherer society. Milk, for example, was consumed only during breastfeeding, and dairy products were completely absent [37]. The first evidence of dairy milk consumption has been dated to about 6,000 years ago [2,38]. Cereals are another interesting example. At the beginning of the Holocene age (12,000–10,000 years ago), cereals—always unrefined—were still rarely eaten. The first evidence of white flour production has been traced back to around 3500 Before Christ (BC) in ancient Egypt [39], but the widespread consumption of highly refined flours began only 150–200 years ago [40].

**Figure 2:**
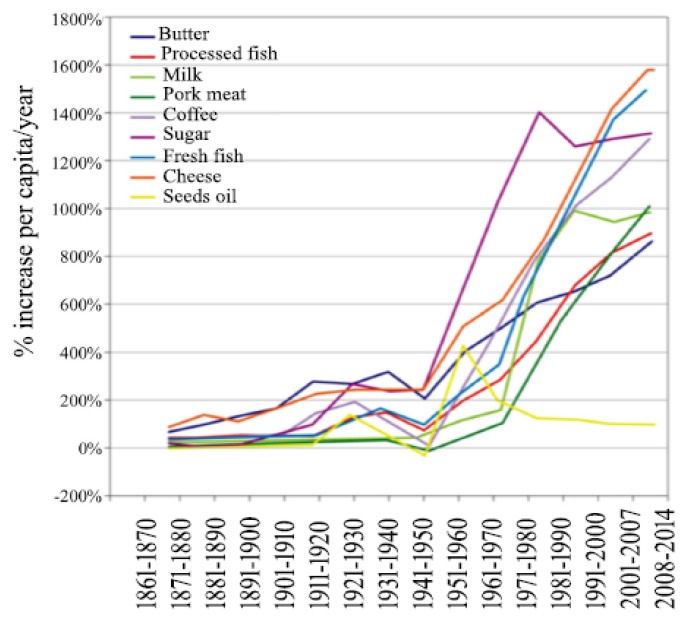
Variation in average food consumption of the population in Italy, one of the countries where the Mediterranean diet was initially discovered, from the end of the 1800s to the early 2000s. The basis of comparison (100%) is the period 1861–1871. Data source: Italian National Research Institute for Food and Nutrition (INRAN), 2013.

The second truly revolutionary change in the human diet was the introduction of industrialized food following the Second World War. Driven by the so-called “green revolution”, which made widespread use of agricultural chemicals, industrialized food production led to a huge rise in the availability of refined cereals, sugar, cheese, butter and coffee (Figure 2). Before this, sugar had been a rare component of the human diet. The first evidence of crystallized sugar can be traced back to 500 BC in India [41]. Before that, the only source of sugar had been honey, available in very limited amounts and only at certain times of the year. Even subsequently, sugar remained a rare commodity. Before the industrial revolution, the annual average sugar consumption in the United States was 6.8 kg per person while in 1970 it exceeded 55 kg, and in 2000 stood at 69 kg [42]. 

Industrialization and the advent of intensive farming also involved a change in farmed animal feed. As a result, the quality of farmed meat, and especially its fat content, is today completely different from the wild animal meat consumed by hunter/gatherers. This is because mammal fat stores are localized in subcutaneous and abdominal adipose tissue in the form of saturated fatty acids (SFAs), while polyunsaturated and monounsaturated fatty acids (PUFAs and MUFAs) prevail in the muscles. It has been estimated that prior to domestication, wild animals consumed most of their subcutaneous and abdominal fat reserves, with the result that our hunter ancestors ate meat with high levels of muscle-stored PUFAs and MUFAs and little or no SFAs [43,44,45,46]. Industrial farming, on the other hand, has led to hyper-fed animals with high percentages of accumulated SFAs [45,46]. 

First extracted around 5,000 years ago, vegetable oils were not initially used as a food. It was only with industrialization and the introduction of new extraction techniques that seed oils became products for human consumption (Figure 2). With the advent of hydrogenation in 1980, hydrogenated vegetable fats suddenly became part of our daily diet. Overall, the increase in dietary fat has certainly had an impact on the microbiota and GI diseases [47]. 

Salt consumption seems to have started at the beginning of the Neolithic age, although its massive consumption only began with the industrial revolution. This has led to an inversion of our ingested sodium/potassium ratio compared to the Neolithic diet. Today, 90% of the salt in the Western-style diet (WSD) comes from processed foods [36]. Food industrialization has also caused a sharp drop in the consumption of fruits and vegetables, and therefore fibers, vitamins and potassium [44,48]. In conclusion, industrialization and the resultant change in diet caused a sudden rise in man’s net acid load after digestion, also known as the Potential Renal Acid Load, PRAL. This is because staple WSD foods like cheese, refined cereals and meat are net acid producers while the vegetable-rich diet of our pre-agricultural ancestors was a net base producer [49]. 

The dramatic change in man’s diet is still ongoing. Ultra-processed foods, recently classified as the NOVA-4 group [50], are now readily available and today account for over 50% of the total energy intake of the United States population [11,51]. 

## 4. The Western-Style Diet and Its Pro-Inflammatory Foods

**Figure 3:**
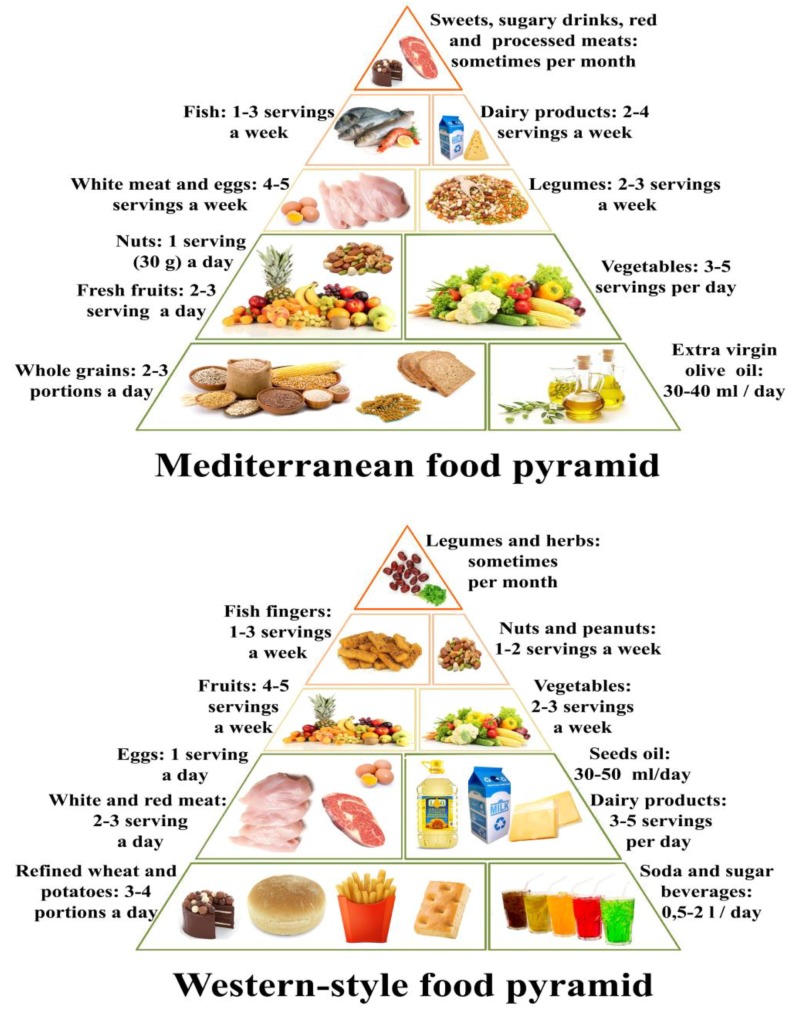
Comparison between the Mediterranean diet (upper) and the Western-style diet (lower) pyramids. The Mediterranean diet pyramid is inspired by the eating habits of Greece, Southern Italy, and Spain before 1950s. The principal aspects of this diet include high consumption of fruits, vegetables, unrefined cereals, legumes and olive oil, moderate to high consumption of fish, moderate consumption of dairy products and low consumption of non-fish meat.

Compared to traditional regional diets, such as Mediterranean, Indian, Japanese and South East Asian, the WSD contains significantly higher amounts of simple refined carbohydrates, saturated fats, red meat, dairy products and industrialized foods, and a concomitantly lower quantity of vegetables, fruits, legumes, whole cereals, raw foods and fibers in general (Figure 3). In fact, the WSD consists mainly of calorie-dense foods high in saturated fats, glycemic carbohydrates and animal proteins. Many studies have looked at the association between WSD and obesity, hypertension [52], chronic kidney disease [53] and other non-communicable diseases [54]. Their findings clearly show that the WSD may promote intestinal inflammation through various mechanisms, including alterations of the microbiota [55,56,57,58]. Although the relationship between the WSD and IBD has been only partially investigated [59,60], it is nonetheless true that the WSD contains nutrients able to elicit a direct or indirect pro-inflammatory effect on the intestine through one of the three components involved in gut physiology: the immune system, the microbiota, and the intestinal barrier. Moreover, the WSD is low in nutrients and micronutrients with anti-inflammatory and anti-oxidant proprieties. It is therefore not unreasonable to consider the transition to the WSD, also defined as diet westernization, as one of the major environmental factors responsible for the rising incidence and prevalence of IBD. A very interesting in-depth study on the effects of this dietary switch on the intestinal microbiota of children has been recently published [61]. The authors clearly demonstrate that the gradual increase of animal proteins (meat and dairy products), saturated fats, and processed and refined foods to the rural vegetarian diet of urban African children drastically changed their microbial profiles and gut functioning. The sharp drop in fiber intake as a result of the dietary change was found to lead to a concomitant fall in microbial populations able to ferment dietary fibers and a sharp rise in other bacterial groups able to metabolize animal proteins, animal fats and sugars. The westernization of the diet has involved and overwhelmed even the traditional oriental diets, such as the Japanese or that of South East Asia, which are diets geographically associated with a low prevalence of IBD [62]. The potential implications of WSD components on the gut immune system, the microbiota and the intestinal permeability will be analyzed in detail in the following sections of this review.

## 5. Carbohydrates

Carbohydrates are classified on the basis of their chemical structure into mono- and di-saccharides (simple sugars, such as glucose, fructose, sucrose), oligosaccharides (fructo- and galacto- oligosaccharides), and polysaccharides (starch, cellulose, inulin). A fraction of the carbohydrates ingested remains undigested in the small intestine and passes to the large intestine to be fermented and/or excreted. For this reason, carbohydrates are also classified according to their absorbability in the small intestine. For example, simple sugars and starches are hydrolyzed and absorbed in the small intestine and are therefore known as *available carbohydrates*. Inulin, fructo-oligosaccharides and galacto-oligosaccharides, known as *resistant starches*, are not hydrolyzed in the small intestine but fermented by the large bowel microbiota, and are therefore called *unavailable carbohydrates*. Finally, insoluble fibers including plant cell-wall polysaccharides, such as cellulose and pectin, and some storage polysaccharides, such as inulin and oligosaccharides, are also called *non-digestible* components because they pass through the digestive tract largely intact, exerting a laxative effect by changing the osmotic properties of the luminal content and by increasing the intestinal fecal mass. The quality of the carbohydrate ingested, for example, whether mono-, di- or oligo-saccharides, also seems to impact the clinical course of IBD. In fact, a correlation has been observed between fructose and lactose malabsorption and IBD severity [59,63]. In light of this evidence, a fructose- and lactose-free diet has been proposed for IBD patients [59].

Lactose malabsorption is frequently detected in IBD, especially in CD patients. However, prevalence is ethnicity dependent, being higher among Asian populations. Moreover, although lactose avoidance may improve symptoms in patients with lactose intolerance, evidence of objective disease improvement is currently lacking. Studies of a lactose-free diet in IBD patients are currently underway [59].

High consumption of fermentable carbohydrates such as glucose, fructose, sucrose, lactose or polyols (sorbitol, mannitol, xylitol and maltitol) has been shown to exceed the intestine’s absorptive ability, increasing dysbiosis and gut permeability, thus promoting inflammation. These fermentable carbohydrates, which include oligo-, di-, and monosaccharides and polyol, are known as FODMAPs (Table 2). Being poorly absorbed, they are rapidly fermented by bacteria in the gut, giving rise to a high osmotic load. This is the rationale behind the FODMAP-exclusion diet. Firstly proposed for Irritable Bowel Syndrome (IBS) patients, the FODMAP-exclusion diet was applied for a limited period to a few IBD patients [64] in an attempt to alleviate their IBS-like symptoms, such as bloating, flatulence, crampy pain, and diarrhea, but proved very limited benefit. Moreover, since undernutrition is a common feature of IBD, the use of this very restrictive diet should be carefully considered [64].

Turning to complex carbohydrates, several epidemiological studies have observed associations between the consumption of refined carbohydrates and the risk of developing IBD. However, these data are not sufficiently statistically significant to allow confirmation of this hypothesis [7]. Nonetheless, the WSD contains many different refined carbohydrates, such as sucrose and starch found especially in soft drinks, white bread, cakes, and desserts, all high-density energy-rich but nutrient-poor products. In the long term, they may cause alterations of the gut microbiota and immune system, increasing the risk for many diseases including IBD [65]. Other dietary approaches have been proposed in an attempt to improve IBD symptoms. These include the “specific carbohydrate diet” (SCD), first described by Haas and co-workers in 1924 to treat celiac disease, and subsequently applied to IBD patients [66,67]. The SCD excludes gluten and complex carbohydrates since they are poorly absorbed and lead to bacterial fermentation, which in turn induces dysbiosis in the gut lumen due to pathobiont expansion (Table 3). Only monosaccharides such as glucose, fructose and galactose are permitted. Clinical trials, albeit involving very few patients, reported improvements in bowel symptoms, with some patients able to discontinue at least one of their IBD medications at the end of the SCD trial period [67].

In conclusion, if poorly absorbed, all carbohydrates may be fermented, promoting dysbiosis and inflammation. An excess of any kind of carbohydrate in IBD patients with intestinal malabsorption may easily exacerbate existing intestinal dyshomeostasis, which in turn may contribute to dysmetabolism of other nutrients, such as fats.

It is therefore reasonable to conclude that IBD diets should carefully monitor the intake of simple and complex carbohydrates and strictly limit lactose, especially in patients intolerant to this sugar. In fact, exclusion of specific carbohydrates (known as carbohydrate monotony) is a common feature of the different diet-based IBD therapies, in light of the suggestion that carbohydrate variation could be linked to IBD pathogenesis [68]. It does, however, seem unlikely that a single sub-group of foods could be the cause of IBD.

## 6. Proteins

Several mechanisms of action have been postulated to explain the possible negative impact of high protein (HP) consumption in IBD patients. One hypothesis is that the intestine’s limited capacity to assimilate proteins, especially in IBD patients, leads to non-absorbed proteins to reach the colonic lumen where they alter microbiota composition by reducing the abundance of bacteria like *Roseburia*/*E.Rectale*, considered health-promoting microorganisms for their ability to produce butyrate [69]. In fact, HP diets appear to induce IBD-like dysbiosis [70]. Rats fed a HP diet showed increased prokaryotic protease activity, a phenomenon apparently linked to the pathogenesis of IBD [71]. HP diets may also lead to an imbalance of metabolites of protein catabolism by colonic bacteria (ammonium, hydrogen sulfide, p-cresol and phenol), which could damage colonocytes and the intestinal barrier [72,73]. Several animal studies on colitis have also demonstrated that proteins derived from red meat, milk and dairy products can exacerbate colitis. However, human epidemiological data only partially support these findings. This is probably due to the complexity of the human diet and diverging nutritional data collection methods [70]. In fact, two epidemiological studies have shown a correlation between IBD and high animal protein intake derived from meat and fish but not from eggs or dairy products, while no correlation was found with the consumption of vegetable proteins [74,75]. Two meta-analyzes conducted in 2011 and 2015 also indicate that the consumption of red, white and processed meat may be considered risk factors for the development of IBD [6,76]. Conversely, two more recent studies have produced conflicting results as to a correlation between meat consumption and IBD activity. The first, by Opstelten et al. [77] involving 165 patients with IBD in remission and 1469 healthy controls, indicated that although IBD patients eat more meat than controls, there was no correlation with disease exacerbation. The second study on 53 patients with active disease and 50 in remission concluded that meat is a risk factor for active disease [77,78]. In addition to the protein metabolism mechanism already discussed, it has also been hypothesized that the effect of meat consumption on the onset and course of these diseases could be linked to the cooking process, which notoriously leads to the formation of carcinogenic or mutagenic molecules such as heterocyclic amines, acrylamide and polycyclic aromatic hydrocarbon, associated with endothelial damage and altered immune responses [79]. However, the most convincing studies on meat and IBD have focused on the risk of high saturated and high n-6 polyunsaturated fatty acid (PUFA) intake as a consequence of high meat (especially red meat) consumption.

Gastro-resistant proteins are another point of attention when structuring an IBD diet since the abundance of gastro-resistant proteins in some foods can alter intestinal permeability. Gluten is a gastro-resistant protein complex. Its digestion gives rise to toxic and antigenic peptides (especially alpha-gliadin peptides), which may not only interfere with the tight junction between enterocytes but also with enterocyte survival, affecting the intestinal barrier. This, together with strong evidence of gluten sensitivity in IBD patients [80], has led to the gluten-free diet being recommended for IBD sufferers. Indeed, a gluten-free diet is often self-imposed by patients in an attempt to alleviate symptoms. Ancient wheat varieties, although seemingly giving rise to similar amounts of toxic and antigenic gluten peptides [81], have been shown to ameliorate gut and systemic inflammation in a range of patients, including IBS sufferers [82]. For this reason, ancient varieties of low immunogenic impact grains, such as Senatore Cappelli (*Triticum turgidum durum*) and Enkir (*Triticum monococcum*), or a novel grain variety with low gluten immunogenicity like Tritordeum [83] should be considered for inclusion in the IBD diet. Being gluten-free, rice has also been suggested as a complex carbohydrate suitable for the IBD diet. This includes whole grain rice, provided it is cooked at length [84].

Milk and dairy products contain large quantities of caseins, gastro-resistant proteins known to increase dysbiosis, inflammation and intestinal permeability in the mouse gut [85]. For this reason, milk and dairy foods are excluded in most IBD diets and indeed, commonly restricted by IBD patients themselves [86]. On the other hand, fermented milk products have shown positive effects on IBD patients. Kefir was administered in a randomized controlled trial to 25 IBD patients with positive effects on clinical parameters, such as erythrocyte sedimentation rate, C-reactive protein, bloating, general-feeling scores, and the *Lactobacillus* bacterial load evaluated in patient feces [87]. Yogurt was administered to IBD patients in a double-blind placebo-controlled clinical trial with beneficial effects on the mean numbers of *Lactobacillus*, *Bifidobacterium*, and *Bacteroides* in the feces of the treated group [88].

In conclusion, although performed with only a few patients, the studies conducted so far would seem to suggest that the IBD diet should reduce the overall amount of meat, eliminate red and processed meat, and eliminate or greatly reduce gluten and dairy products (caseins), with the sole exception of yogurt and kefir.

## 7. Fats

Recent epidemiological data attribute a protective effect to n-3 PUFAs in UC prevention and therapy [89,90]. Conversely, consumption of higher ratios of n-6/n-3 PUFAs has been associated with increased UC incidence. The role of n-3 PUFAs in CD prevention and therapy, however, appears complex and controversial [89]. The physiological basis for PUFAs’ role in IBD is that the eicosanoids produced by n-3 and n-6 PUFA are key immune response regulators, being precursors of several pro-inflammatory (n-6 PUFA) and anti-inflammatory (n-3 PUFA) molecules. It has been demonstrated that n-6 PUFAs are more available than n-3 PUFA for eicosanoid metabolism in the UC colon mucosa. For this reason, dietary supplementation with n-3 PUFA, and especially eicosapentaenoic acid (EPA), can reduce the production of prostaglandin E2, thromboxane B2 and hydroxyeicosatetraenoic acid inflammatory mediators [91,92,93]. The anti-inflammatory effects of n-3 PUFA have also been linked to the synthesis of molecules like resolvins, protectins and maresins, which counteract IBD-related dysbiosis [94], and the down-regulation of pro-inflammatory genes [95]. However, two IBD studies have indicated that PUFA intake may have a different impact in CD compared to UC patients. In their study on 137 UC patients and 38 healthy controls, Wiese and collaborators [96] showed that the concentration of inflammatory tissue cytokines in UC patients was directly related to the amount of serum SFAs and inversely related to total PUFAs, EPA and docosahexaenoic acid (DHA), confirming the protective role of n-3 PUFAs in UC. But a recent study by Scoville and collaborators [97] on 116 CD patients and 27 healthy controls showed that n-3 PUFA serum concentrations were directly related to disease severity. In particular, the authors found EPA concentration to be strongly related to pro-inflammatory serum cytokine concentrations. This datum not only suggests that fatty acid dysmetabolism occurs in patients with CD but also that n-3 PUFA and EPA serum levels may also have a negative effect in CD patients. Conversely, no correlation with SFA concentration was observed in UC patients [97]. Interestingly, both studies, which administered the same amounts of meat, total fat, and SFA to IBD patients and healthy controls, found that n-6 PUFA/n-3 PUFA ratios were similar in CD patients and healthy subjects, despite higher respect the WHO recommendations. 

In summary, we may conclude that high fat intake should be avoided in IBD sufferers since high fat diets may lead to an accumulation of secondary bile acids, such as deoxycholic acid, which can inhibit Bacteroidetes and Firmicutes phyla growth, leading to IBD-like dysbiosis [7]. However, a prospective study carried on 170,805 women showed no correlation between fat consumption and IBD risk, but rather a positive correlation between high trans-unsaturated fat intake and UC risk [89]. In addition, the intake of n-3 PUFA and EPA in particular, within foods or supplements, may have positive effects on health, but only for UC patients. 

## 8. Dietary Fibers

Dietary fibers are non-digestible complex carbohydrates present in plants. They are grouped according to their solubility or insolubility in water. Since humans lack fiber-specific enzymes, fibers remain mainly undigested and are therefore not absorbed by the gastrointestinal tract. However, small amounts of the ingested fibers are fermented by the bacteria in the gut, especially in the colon. Present in fruits, vegetables, cereals and legumes, dietary fibers can exert a protective role against IBD through four independent mechanisms [90]:

Reducing intestinal transit time;

Increasing the synthesis of SCFAs such as acetate, butyrate and propionate, which may positively modulate intestinal inflammation;

Improving gut microbiota composition, thus counteracting IBD-related dysbiosis;

Contributing to the maintenance of intestinal barrier functions.

No conclusive studies exist evidencing the efficacy of dietary fibers in slowing IBD progression [98]. A study conducted by Andersen and collaborators [90] showed no association between average fiber intake (total fiber, fiber from fruit, vegetables and cereals) and the subsequent development of CD or UC. Moreover, it has been shown that fibers may exacerbate gastrointestinal (GI) symptoms, such as abdominal distension, flatulence, constipation, and diarrhea in IBD patients during remission periods [99]. It is therefore reasonable to postulate that a high fiber diet is not beneficial for individuals with insufficient “fiber-degrading bacteria” in their gut microbiota [100]. However, while this type of diet may be contraindicated for patients during disease flares, it is nonetheless highly recommended, with appropriate adjustments, after remission in an attempt to approximate the 30g/die WHO recommended intake. 

As suggested by Eswaran and collaborators [99], patients in remission should receive a diet with low levels of insoluble fibers, such as those in skinned vegetables like zucchini, carrots, eggplants, green beans and chards. The vegetables should be very well cooked and consumed as a cream, at least initially. Peeled fresh fruit is another option, providing small quantities of insoluble fiber. Putting ingredients through a juice extractor will further reduce the amount of insoluble fiber present in fruit, vegetables and legumes while maintaining most of the soluble fiber fraction, vitamin and mineral content. There is also some unsubstantiated evidence that fruit and vegetable consumption may have a protective immune-modulating effect on IBD patients since they contain protective compounds that may help to prevent disease flares [101]. Another source of insoluble fibers is whole grain cereals. Here too, it seems appropriate to include cereals in the diet only after remission, doing it gradually to avoid worsening of the disease-related GI symptoms [84,99]. Although many IBD patients avoid foods containing fibers, this may lead to micronutrient deficiency and aggravate the malnutrition often presented by sufferers of this disease.

## 9. Micronutrients

IBD patients tend to suffer from nutrient malabsorption and/or have erroneous nutritional patterns [102], with the result that many are likely to present micronutrient, vitamin and mineral deficiencies. Since micronutrient deficiency may affect immune system physiology and thus facilitate disease relapse, diet or supplementation adjustment strategies could be a key to improove patients’ quality of life [103]. The main vitamin deficiencies found in IBD-patients are of fat-soluble vitamins A (retinol and retinoids) and D, and water-soluble vitamins B12 and B9 (folic acid). Experimental models have provided some evidence that vitamin A (provided as retinol) supplementation attenuates intestinal inflammation and improves the gut environment, with significant benefits to health [65]. As antioxidants, vitamins A have a probable protective role against free radicals and oxidative damage, both of which are increased in these patients [104].

As well as regulating bone, calcium, and phosphorus metabolism, Vitamin D has a key role in intestinal defense mechanisms, regulating the adaptive and innate immune systems and suppressing microbial invasion of the epithelium [105,106]. Vitamin D deficiency has been detected in 16% to 95% of IBD sufferers, mainly in CD patients [107]. This may be due to several factors, such as intestinal malabsorption, low exposure to sunlight, lack of physical activity, or insufficient dietary intake. Therapy targeting the vitamin D3 signaling pathway has been suggested in IBD since it may regulate both innate and adaptive immune functions. However, the overall effect of vitamins on IBD is still not well understood, and the clinical studies performed have shown contrasting results. A Danish study conducted between 2005 and 2008 randomized 94 IBD patients to receive oral vitamin D3 or placebo. Although patients receiving vitamin D3 showed a reduced risk of clinical relapse (from 29% to 13%), this was not statistically significant [108]. Similarly, a more recent Iranian study on 108 IBD patients found that oral vitamin D3 supplementation gave rise to lower serum TNF levels, which, however, were also not statistically significant [109]. 

Folic acid, or folate, is available in several vegetables and is absorbed in the duodenum and proximal jejunum. Both micronutrients are essential for cell metabolism, and folate deficiency is often correlated with vitamin B12 deficiency, this latter mainly available in animal products. Despite their ample availability in food, folate and vitamin B12 absorption occurs in areas often damaged by phlogosis, which may be the cause of impaired uptake in IBD-patients [110].

Trace elements are another important avenue of ongoing research into the prevention and control of IBD. Iron deficiency is very frequent in IBD and the leading cause of anemia among IBD patients. In a recent study, 27% of all CD and 21% of all UC patients were found to have anemia [111]. An iron-, heme- and nonheme-rich diet or oral/parenteral supplementation is therefore mandatory. However, iron—and especially ferrous sulfate—is a known pro-oxidative agent, raising concern that oral iron may increase oxidative stress in patients with colitis [112]. 

Zinc is an essential micronutrient, an enzyme co-factor involved in cellular immunity, cell growth and wound healing. Zinc deficiency has been associated with excessive loss of GI secretions due to chronic diarrhea or fistula drainage in IBD patients. Prevalent in 15% to 40% of IBD patients, it has also been associated with hospitalization, surgery and other disease complications [113].

Selenium is a non-metal trace element existing in both the inorganic and organic form. It is translationally incorporated as selenocysteine into selenoproteins, which can modulate various cellular pathways involved in inflammation. Adequate selenium intake is thus essential for normal immune functions [114]. In fact, selenium deficiency has been correlated with gastrointestinal inflammation and altered gut microbiota composition [115]. Epidemiological studies suggest that selenium serum levels are inversely correlated with IBD incidence. Moreover, selenium blood concentrations were found to be significantly lower in Caucasians and Asiatic IBD patients, especially in CD [116,117]. Although further studies are necessary to assess the mechanism underlying the effect of selenium deficiency in IBD, it would seem important to ensure adequate selenium intake in all IBD patients. 

## 10. Food Additives and Ultra-Processed Food

Ultra-processed foods (NOVA 4 classification) are the result of a series of industrial processes, many requiring sophisticated equipment and technology. Ultra-processed food manufacture entails fractioning whole foods into different substances, their chemical modification and subsequent assembly with other modified and unmodified food products. The industrial techniques used include extrusion, molding, pre-frying, and the addition of “cosmetic” palatability additives [50]. The presence of high levels of ultra-processed food in the diet has already been linked to a number of non-communicable diseases including cancer, obesity and type 1 and 2 diabetes [118,119,120]. Despite this, however, the role of these foods on the intestinal microbiota, and on IBD in particular, has never been investigated. However, there is well-documented scientific evidence of the negative effects on the microbiota of food additives typically present in a considerable number of ultra-processed foods. Various studies have clearly shown the negative effect of non-caloric artificial sweeteners often present in carbonated soft drinks on microbiome composition and functioning [121]. The sweetener sucralose has been shown to reduce the relative amount of *Clostridium* cluster XIVa in mice feces [122]. Dietary emulsifiers have also been shown to directly alter human microbiota composition and gene expression in an ex vivo model [123], while maltodextrins, commonly used for the production of soft drinks, candy and sport energy products, have been found to cause microbiota alterations [124] and affect gut epithelial cells, reducing mucus production and exacerbating gut inflammation [125].

The commonly used food additive carrageenan has been shown to induce intestinal inflammation in animal models. Although there is little evidence of the role carrageenan may play in the development of IBD since direct carrageenan assessment trials in man are unethical, animal studies have clearly demonstrated that this additive induces IBD-like histopathological features, alters the microbiome, and disrupts the intestinal epithelial barrier [126]. Red meat, eggs naturally contain high levels of sulfites due to their high cysteine content, which can become a substrate for the production of hydrogen sulfide (H_2_S) by sulfate-reducing bacteria. However, sulfites are also abundantly used as food additives to limit bacterial contamination. Generally considered safe for human consumption, they are present in food and alcoholic beverages in quantities up to 5000 parts per million (ppm). However, even at lower concentrations, sulfites have been shown to damage beneficial bacteria, such as *Lactobacillus* and *S. thermophilus* in the human gut [127]. Sulfites can be produced by intestinal microbiota even starting from taurine (from the bacterium *B. wadsworthia*) which is a typical ingredient of energy drinks or from chondroitin sulfate, a widely used food supplement in the treatment of osteoarthritis [128]. Some studies have found that patients with UC show a higher production of H_2_S in the gut, but it is not clear whether this increased synthesis is simply linked to the increased intestinal inflammation [128].

## 11. Alcohol

Small quantities of alcohol are first metabolized in the stomach. Larger amounts are absorbed in the small intestine and may reach the large bowel from the bloodstream where it is metabolized by luminal bacterial alcohol dehydrogenase, producing the toxic compound acetaldehyde [129,130]. Severe dysbiosis has been associated with chronic alcohol abuse. In addition, the high sugar content of some alcoholic drinks may be associated with osmotic diarrhea [131]. A study of 52 Crohn’s and 38 UC patients with an alcohol consumption pattern similar to the general population found that although 75% of the IBD patients reported worsening of their symptoms, this could not be related to alcohol intake [132]. In 2018, a prospective cohort study of 262,451 subjects enrolled in six countries showed no association between alcohol use and the onset of IBD [133]. Interestingly, two studies have found a correlation between UC disease activity and sulfite, an additive commonly used also in processed alcoholic drinks [134,135]. In conclusion, although there are insufficient data to define alcohol as a risk factor for IBD, its toxic effect on colon mucosa strongly suggests limiting alcohol intake in IBD sufferers. 

## 12. Nutritional approach in IBD

Recently, numerous new dietary philosophies have become popular. These include the paleo and the vegan diets, often recommended in the management of gastrointestinal diseases such as IBD. However, there is no clinical evidence to support their use in IBD [113]. The only nutritional approach with strong evidence of efficacy is Exclusive Enteral Nutrition (EEN), a diet based on foods classified according to their nitrogen content as: elemental (amino acid based), semi-elemental (oligopeptide based) and polymeric (whole protein based). EEN has proved as efficacious as steroid treatment in inducing remission of CD in children and adults but not of UC [136]. Although the above-mentioned low FODMAPs diet has been mainly used to alleviate symptoms in IBS patients, there are currently no consistent data supporting its use in IBD. A pilot study conducted on 52 CD and 20 UC patients showed that 50% of patients had an improvement in abdominal pain, bloating, and diarrhea [137]. A double-blind controlled placebo cross-over trial was conducted with 32 patients with quiescent disease and IBS-like symptoms that had proved responsive to a Low-FODMAP diet. Patients were randomly assigned to receive fructans, galacto-oligosaccharides, sorbitol, glucose or placebo. The study found that only fructans exacerbated symptoms in patients, which casts into question the real usefulness of the low FODMAPs diet in IBD [138]. Another uncontrolled clinical trial included a semi-vegetarian diet as an “add-on therapy” to the biological drugs used to induce remission. 26 adults and 11 children with recently diagnosed CD and 9 adults with relapsing CD received a lacto-ovo semi-vegetarian diet that included fish once a week and meat once every 2 weeks. Results showed an increase of the expected remission rate in all patients [139]. Yet another study of CD patients on a semi-vegetarian diet for two years reported the absence of CD relapse in 15 of the 16 patients treated [140]. The IgG exclusion diet, on the other hand, is a personalized regime based on preliminary blood titration of food IgG. Two studies conducted by the same research group have demonstrated the efficacy of this diet. The first, an open-label pilot study, was conducted on 40 symptomatic CD patients [141] while the second was a randomized study enrolling 76 subjects with active CD. In this second study, the foods most frequently excluded were milk, beef, pork and eggs. The personalized diet significantly improved patients’ quality of life and disease activity, measured using the Crohn’s Disease Activity Index (CDAI) and Harvey-Bradshaw Index (HBI) [142]. 

Although SCD diet is one of the most popular among IBD patients, it has many restrictions making compliance difficult. Obih and collaborators [143] conducted a retrospective study on 26 IBD children who had followed the SCD for 3–48 months, 18 of whom were receiving concomitant medication therapy. 12 of the 26 patients showed improvements in their clinical and inflammatory markers. It should, however, be noted that in the medium and long term the SCD diet may cause adverse effects, such as weight loss and nutritional deficiencies. Patients following this diet must therefore be carefully monitored.

Another study on the SCD, involving 50 adult IBD (mainly colonic CD) patients, obtained a better symptom score and a higher quality of life. Some patients were even able to maintain clinical remission without medication [144]. The fecal microbiota of patients on the SCD was observed to have a higher biodiversity index compared to matched patients eating a WSD [144]. These data suggest that the SCD may be a good starting point from which to plan a dietary regime for IBD patients, especially in the maintenance phase.

## 13. Conclusions

Although the study data are still often unsubstantiated, it is important not to underestimate the role diet can play in supporting conventional IBD therapies, not only helping to induce and maintain clinical remission, but above all, improving patient quality of life. 

While controversy still surrounds the exact role played by diet in the development of IBD, there are indications that diet may significantly modulate disease onset and activity. No formal dietary guidelines exist, however, for patients with IBD. The wide variability of study results is due in part to the design limitations of what are largely underpowered, retrospective studies, and in part to the heterogeneous disease subtypes (UC, CD) recruited to the studies.

The complexity of the human diet makes it very difficult to identify individual foods as risk factors. It is in fact more likely that complex nutritional patterns very different from either the traditional Mediterranean diet or other traditional diets matching our evolutionary status may impact pathogenesis and the clinical course of IBD. In evolutionary terms, in fact, people in industrialized countries can be described as nutritionally mismatched and more susceptible - to degrees depending on their genetic background - the many non-communicable diet-related diseases, including IBD.

**Figure 4:**
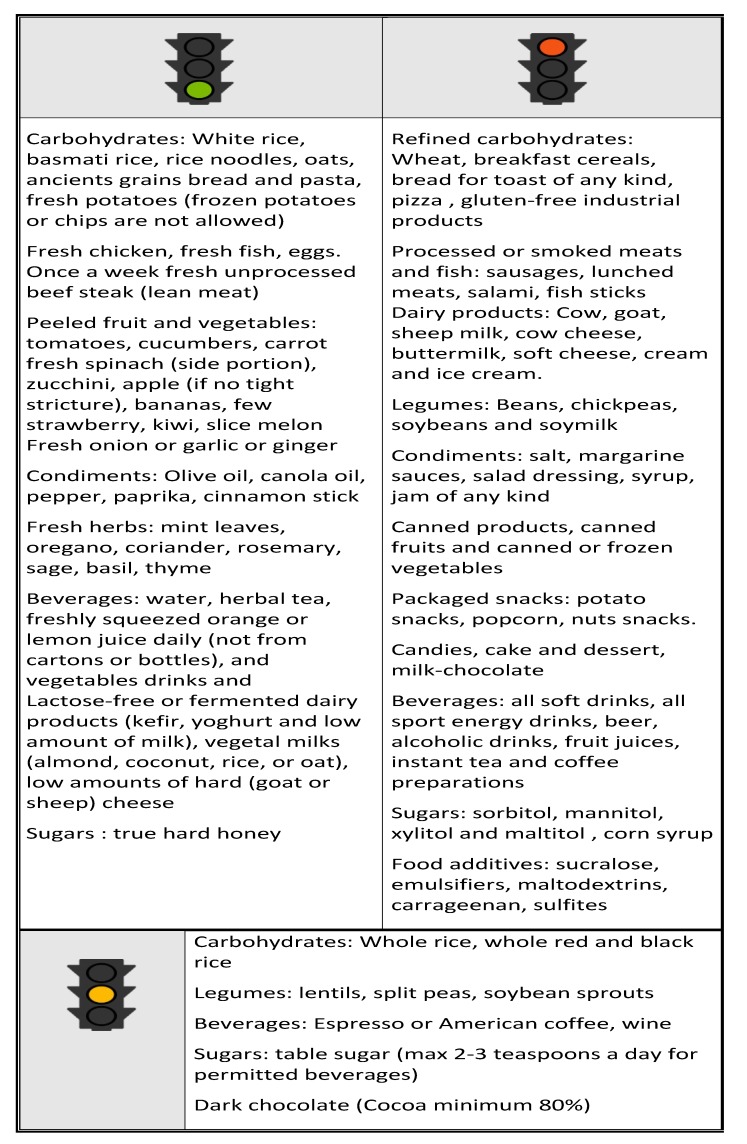
A possible diet for IBD: dietary foods and factors potentially affecting positively or negatively host intestinal barrier, immunity and microbioma in IBD patients.

We now know the mechanisms of action whereby certain food groups may have a negative impact on the microbiota, triggering intestinal inflammation or intestinal permeability. We also know that the Western population tends to eat excessive amounts of “dangerous” foodstuffs. As mentioned above, the reason why studies have failed to provide strong evidence of dietary efficacy could be due to individual variability as well the small patient series studied. However, as one of the factors involved in the onset and progression of IBD, diet requires further in-depth study, especially since IBD patients tend to have disordered and unbalanced eating patterns. Although guidelines on IBD prevention and treatment are starting to appear (Figure 4) [60], it must be remembered, however, that they are based more on logical assumptions and animal models than on evidence provided by broad controlled human clinical studies.

## Figures and Tables

**Table 1:** The multifactorial role of the SCFAs in energy homeostasis in host immune function and host physiology [25,27,28]. GABA, gamma-Aminobutyric acid; CNS, Central Nervous System; ENS, Enteric Nervous system; PYY, Peptide YY; GLP-1, Glucagon-like peptide-1.

CNS Activity	Metabolic Regulations	Immune Homeostasis
GABA production in CNS Modulation of ENS and CNS function Serotonin biosynthesis	Energy source Intestinal gluconeogenesis Antilipolytic activity (WAT) Lepitin production (WAT) Release of major appetite and glucose regulatory peptides (PYY, GPL1) (L-cell)	Enhancement of IL-10 (DC) IL-18 biosynthesis (IEC) Treg polarization Reduction of TNF, IL-6, IFN (DC) IEC barrier fortification IgA promotion

**Table 2:** Carbohydrates included or excluded in the low-FODMAPs diet.

Food Group	Include	Exclude
Fruit	Bananas, strawberry, raspberry, blueberry, orange, mandarin, clementine, cantaloupe, grapes, melons, lemon, lime, kiwi, passion fruit	Apple, applesauce, apricots, blackberries, cherries, nectarines, pears, peach, plum, prune, watermelon, grapefruit, dried fruit
Vegetables	Carrots, celery, corn, alfalfa, bean sprouts, bell pepper, broccoli (<1/2 cup), bok choy, cucumber, eggplant, green bean, kale, lettuce, potato, spinach, spring onion (green top), squash, tomato, turnip, zucchini	Brussels sprouts, asparagus, avocado, beetroot, cauliflower, cabbage, garlic, leek, mushroom, onion, pea shallot, snow pea, sweet corn, sweet potato
Grains	Rice, oats	Wheat, rye
Legumes	Certain legumes (soya)	Many legumes (chickpeas, lentils, beans)
Dairy	Lactose-free yoghurt and milk; almond, coconut, rice or soy “milk”, hard cheese, low-lactose cheese	Cow, goat, sheep milk, buttermilk, soymilk, soft cheese cream and ice cream
Beverage	Fruit juice and vegetable juices from permitted foods, wine	Soft drinks, sports drinks, fruit juice and vegetable juices from unpermitted foods, alcohol
Other	Maple syrup	Honey and sweeteners

**Table 3:** Carbohydrates included or excluded in the Specific Carbohydrate Diet (SCD).

Food Group	Include	Exclude
Fruit	All fresh fruit	Canned fruit (because of possible added sugars and starches)
Vegetables	Fresh vegetables	Canned or frozen vegetables (because of possible added sugars and starches), potatoes and yams
Grains	None	All cereal grain
Legumes	Certain legumes (lentils, split pea)	Certain legumes (chickpeas, soybeans) and soybean “milk”
Dairy	Lactose-free dairy (milk, cheese and yogurt)	Milk and all dairy products
Beverage	Wine	Instant tea, coffee and beer
Other	Honey,	Corn syrup, chocolate
